# COVID-19 Testing in a Weekly Cohort Study of Gay and Bisexual Men: The Impact of Health-Seeking Behaviors and Social Connection

**DOI:** 10.1007/s10461-022-03831-1

**Published:** 2022-09-01

**Authors:** Mohamed A. Hammoud, Nathanael Wells, Martin Holt, Benjamin Bavinton, Fengyi Jin, Lisa Maher, Steven Philpot, Bridget Haire, Louisa Degenhardt, Adam Bourne, Peter Saxton, Phillip Keen, Daniel Storer, Garrett Prestage

**Affiliations:** 1 Kirby Institute, UNSW Sydney, Level 6, Wallace Wurth Building UNSW, 2052 Sydney, Australia; 2 Centre for Social Research in Health, UNSW Sydney, Sydney, Australia; 3 National Drug and Alcohol Research Centre, UNSW Sydney, Sydney, Australia; 4 Australian Research Centre in Sex, Health and Society, La Trobe University, Melbourne, Australia; 5 Department of Social and Community Health, School of Population Health, University of Auckland, Auckland, New Zealand

**Keywords:** COVID-19 testing, Coping during COVID-19, Gay, bisexual, and other men who have sex with men (GBM), Gay community, Severe acute respiratory syndrome coronavirus-2 (SARS-CoV-2).

## Abstract

Gay, bisexual, and other men who have sex with men (GBM) have developed community norms for regular HIV/STI testing. We investigated factors associated with self-reported COVID-19 testing in response to reported COVID-19 cases and public health restrictions. Participants responded to weekly cohort surveys between 10th May 2021 and 27th September 2021. We used the Andersen-Gill extensions to the Cox proportional hazards model for multivariable survival data to predict factors influencing COVID-19 testing. Mean age of the 942 study participants was 45.6 years (SD: 13.9). In multivariable analysis, GBM were more likely to report testing during periods of high COVID-19 caseload in their state of residence; if they were younger; university educated; close contact of someone with COVID-19; or reported coping with COVID-19 poorly. COVID-19 testing was higher among men who: were more socially engaged with other GBM; had a higher proportion of friends willing to vaccinate against COVID-19; and were willing to contact sexual partners for contact tracing. Social connection with other gay men was associated with COVID-19 testing, similar to what has been observed throughout the HIV epidemic, making community networks a potential focus for the promotion of COVID-19 safe practices.

## Introduction

In December 2019, the novel coronavirus (COVID-19) was identified [1]. With no effective treatments or vaccines at the time, countries began implementing control and prevention measures, including physical distancing, to minimize the impact of COVID-19 [2]. From March 2020, Australian jurisdictions introduced public health measures to slow case numbers and minimize the impact on healthcare systems [3]. These included physical distancing measures, limitations on social gatherings, working from home where possible, and heightened hygiene measures [4, 5].

Decreasing rates of COVID-19 throughout April and May 2020 saw a gradual easing of restrictions across Australia. Subsequent local outbreaks precipitated sustained lockdown periods lasting more than 7 days: Melbourne in the state of Victoria (August-October 2020), Sydney’s Northern Beaches district in the state of New South Wales (December 2020-January 2021); Sydney (June-October 2021); and Melbourne (July-November 2021) [6, 7]. In response to these outbreaks, heightened restrictions were introduced. These included restricting travel to within five kilometers (3.1 miles) of one’s residence, bans on household visitations, the closure of non-essential retail services, and nightly curfews. As smaller outbreaks occurred in other Australian jurisdictions, similar control measures were replicated. While “singles bubbles” allowed individuals to visit one other person outside of their household, COVID-19 restrictions strongly recommended people avoid casual sex and meeting new sexual partners [8].

COVID-19 lockdown measures negatively impacted community networks, social venues, the mental wellbeing of gay and bisexual men (GBM) and made it more difficult to maintain mutually supportive relationships [9]. Among GBM, lockdown measures were associated with a reduction in the number of sexual partners [10–12]. Lockdown measures were accompanied by reductions in the use of HIV pre-exposure prophylaxis (PrEP) [11, 13, 14] and HIV testing [14].

Community connectedness has been shown to support resilience and health-related help-seeking behaviors in both marginalized populations and during the COVID-19 pandemic [15, 16]. Gay community engagement has historically been a key factor in HIV prevention and testing, with regular testing for HIV having become normalized among GBM [17, 18]. Peer networks among GBM play an important role in disseminating information and normalizing new prevention technologies, in the same way they do routine HIV testing [19]. This entrenched sense of community responsibility regarding personal and public health may shape how GBM engage with COVID-19 testing.

By November 2021, over 45.6 million (179 per 100,000 people) COVID-19 tests had been performed in Australia with an average of 75,000 tests per day. Daily testing fluctuated from the lowest on April 16th 2020 (7,124 tests) to the highest on April 26th 2021 (252,134 tests) [6, 7]. Whereas monitoring of HIV testing has been a key component of HIV surveillance among Australian GBM [20], no monitoring of COVID-19 testing patterns in this population has been reported. We investigated how GBM, for whom regular testing for HIV may be normalized, may have adopted similar strategies in relation to COVID-19 testing.

In this paper, we investigate factors associated with self-reported COVID-19 testing within a cohort of Australian GBM. Using weekly survey data from a cohort study, we also describe a method of rapid data collection to monitor near real-time changes in behaviors to new COVID-19 cases and public health restrictions.

## Methods

### Study Design

The *Following Lives Undergoing Change (Flux) Study* is an online, prospective observational study on the health of GBM in Australia [21]. Launched in 2014, *Flux* was an open cohort study which collected biannual survey data. In early 2020, *Flux* was adapted to explore the impact of COVID-19 physical distancing restrictions. This reorientation was referred to as the *Flux COVID-19 Online Diary.*

We predicted factors associated with COVID-19 testing would be similar to factors associated with HIV testing among GBM [17, 18] including levels of risk and/or exposure, social engagement, and testing norms among peers [22–24]. Adapting methods used for the *Flutracking* online surveillance study [25], we implemented a weekly online cohort to monitor and report individual changes in sexual behaviors, HIV prevention strategies, HIV testing, and COVID-19 testing and vaccination throughout the COVID-19 pandemic. The *Flux Study* and the *Flux COVID-19 Online Diary* study protocol and all supporting documentation were approved by the Human Research Ethics Committee of the UNSW Sydney (HC14075 and HC200286, respectively).

### Eligibility and Recruitment

Eligibility to participate in this study included: self-ascribed male gender, aged 16 or above, residence in Australia, sex with a male partner in the previous twelve months, or self-identification as gay, bisexual, or otherwise same sex attracted. Participants in the existing *Flux Study* were invited to take part in the *Flux COVID-19 Online Diary*. Paid advertisements were distributed through Facebook and Instagram, and community partner organizations promoted the study through their social media networks and newsletters.

### Instruments

All participants were asked to complete a baseline COVID-19 survey during April 2020 and, thereafter, the weekly *Flux COVID-19 Online Diary.* The baseline survey took approximately 20–30 minutes to complete and consisted primarily of forced-choice answers and a small number of free-text questions. The weekly *Flux COVID-19 Online Diary* usually took less than five minutes to complete and consisted of both forced-choice and free text questions.

## Procedure

### Obtaining Consent

Participant confidentiality was maintained at all times. Consent was obtained through an online electronic consent process [21]. Participants were required to confirm their understanding of all study requirements, their willingness to participate in weekly surveys, and to provide an email address to receive weekly reminders to complete their survey. The database automatically generated a deidentified participant ID and individualized links removed the need for direct researcher contact. Participants could withdraw their consent from the study at any time via an automated link that was included in all email communications.

### Weekly Diary Invitations

Continuing participants completed their baseline COVID-19 survey during April 2020 and new participants did so upon enrolment. The weekly *Flux COVID-19 Online Diary* commenced on May 10th 2020. After 52 weeks of consecutive weekly surveys, data collection periods became quarterly with subsequent data collection points in late June and late September 2021.

Participants were sent an email containing an individual, deidentified URL link to their weekly *Flux COVID-19 Diary* every Sunday at 10:30am and were asked to complete their entry within 48 h. All participants who did not complete their diary entry were automatically sent an email reminder on the following Monday at 10:30am. To encourage retention, participants who completed their diary entry within 48 h were automatically given the chance to win weekly prizes worth $AUD200.00.

### Measures

The baseline survey included: demographic characteristics, age, country of birth, state and suburb of residence, sexual orientation and identity, education, current employment, HIV status, use of antiretroviral drugs as either treatment or PrEP, sexual behaviors, HIV risk behaviors, and COVID-19 knowledge and beliefs. Social engagement with gay men was asked at baseline [18]. As there was little literature on COVID-19 testing among GBM at the time of study design, selected measures were informed by literature on HIV and STI testing, risk behaviors, and prevention among GBM [22–24]. Participants were asked to report on three broad health related areas: HIV; mental health; and COVID-19.

Each week, GBM would report if they tested for COVID-19, and whether the test was a nose or throat swab or a blood test. These data were reported prior to the availability of rapid antigen tests and when COVID-19 testing was primarily administered by healthcare workers on-site through polymerase chain reaction tests. Weekly questions about COVID-19 included: vaccination, contact with someone diagnosed with COVID-19, and willingness to contact non-relationship sexual partners after potential exposure, all with reference to the previous seven days.

Additional questions were included in the final two diary entries which asked men to report the proportions of their gay friends who had avoided social events and intended to get vaccinated using three categories (none, a few, most or all). Weekly mental health questions included: a 6-point Likert scale measuring self-assessed coping with COVID-19 restrictions (responses ranged from coping very poorly to very well); and social connection with family and friends in the previous seven days.

### Statistical Analysis

Statistical analysis was conducted using Stata version 14 (Stata Corp, Texas, USA). Baseline characteristics were summarized by COVID-19 testing history (never tested for COVID-19 vs. any test of COVID-19 during the study period) and expressed as numbers and percentages. Categorical variables were analyzed using Pearson’s chi-square test, and continuous variables were analyzed using t-test. We used Type I error of 5% for these analyses. Longitudinal analyses included all men who provided at least one follow-up diary. Participants with missing data included in this analysis were noted accordingly and included in the denominator.

As COVID-19 testing was a recurrent event throughout the study period, we used the Andersen-Gill (AG) extensions to the Cox proportional hazards model to determine associations with COVID-19 testing and recurrent testing. Incidence of event was defined as any report of COVID-19 test performed during the study period following baseline survey. COVID-19 testing mandates usually prescribed retesting two weeks after an initial test [26], so subsequent tests within two weeks were not included as an incident event in the analysis. Follow-up time started 7-days prior to the date when participants first responded to the weekly survey and ended at the date they last completed the weekly survey during the study period. Variables measured within these recall periods capture recent behaviors that were likely associated with their decision to test for COVID-19 [27]. We assessed a range of demographic variables, COVID-19 related measures (e.g. contact with someone who had COVID-19), items on mental health, and factors previously found to influence HIV testing such as community connectedness and sexual behaviors [22–24]. Bivariable associations with p < 0.050 were included in the multivariable model to avoid the omission of relevant variables. On multivariate analysis, p < 0.050 was considered the cut-off for a significant difference with all variables included in the model. Non-significant variables were retained in the model. We presented adjusted hazard ratios (aHR) and 95% confidence intervals (CI).

## Results

### Enrolment Phases

In May 2020, 718 of the 913 participants (78.6%) who participated in the *Flux Study Covid-19 baseline survey* in April 2020 enrolled in the *Flux COVID-19 Online Diary* study. Respondents who did not join the study were younger (mean: 42.0 vs. 45.6; p = 0.001) and less likely to be university educated (68.3% vs. 76.4%; p = 0.006). They were otherwise similar in terms of sexual identity (p = 0.250), state of residence (p = 0.435), HIV status (p = 0.370), and country of birth (p = 0.189).

Between June 22nd 2020 and March 1st 2021, 224 new participants joined the study bringing the total diary sample size to 942. Compared to the 718 men recruited prior to 2020, the 224 men recruited in 2020 were less likely to be born in Queensland (6.7% vs. 13.9%; p = 0.019); more likely to be born overseas (4.3% vs. 10.7%; p = 0.004); and report an HIV positive serostatus (17.9% vs. 8.1%; p < 0.001). They were otherwise similar in age (p = 0.671), education (p = 0.396), and sexual identity (p = 0.082).

Over the 54-week study period and accounting for date of first enrolment, 17.4% (N = 164) of participants completed 100% of their diaries, 19.4% (N = 183) completed between 91% and 99%, 14.4% (N = 136) completed between 51% and 90%, 32.6% (N = 307) completed more than 1 diary and up to 50%, and 16.1% (N = 152) only completed 1 diary.

### Sample Characteristics

The mean age of GBM included in this sample was 45.6 years (SD: 13.9). Most were born in Australia (79.0%), identified as gay (89.6%) or bisexual (5.4%), and most were university educated (71.9%; Table 1). Most participants lived in Australia’s two most populous states, New South Wales (45.6%) and Victoria (26.3%), and one in ten men (10.4%) reported living with HIV.

**Table 1:** Sample characteristics (N = 942)

N (%)	No history of COVID-19 testing 523 (55.5)	Any history of COVID-19 testing 419 (45.5)	p value
**Recruitment source**			0.946
Social media	282 (53.9)	234 (55.8)	516 (54.8)
Gay community organisations	33 (6.3)	28 (6.7)	61 (6.5)
Gay apps and websites	85 (16.3)	68 (16.2)	153 (16.2)
Personal networks	32 (6.1)	24 (5.7)	56 (5.9)
Consent from other study	47 (9.0)	37 (8.8)	84 (8.9)
Other	44 (8.4)	28 (6.7)	72 (7.6)
**Age Mean (SD)**	46.08 (14.00)	44.99 (13.84)	0.237
**Country of birth**			0.071
Australia	405 (77.4)	339 (80.9)	744 (79.0)
New Zealand, Europe & North America	55 (10.5)	45 (10.7)	100 (10.6)
Asia	23 (4.4)	20 (4.8)	43 (4.6)
Other	40 (7.6)	15 (3.6)	55 (5.8)
**State of residence**			0.001
New South Wales	230 (44.0)	200 (47.7)	430 (45.6)
Victoria	121 (23.1)	127 (30.3)	248 (26.3)
Queensland	72 (13.8)	43 (10.3)	115 (12.2)
Other states	100 (19.1)	49 (11.7)	149 (15.8)
**Employment**			0.014
Full time	28 (55.1)	270 (64.4)	558 (59.2)
Part time	71 (13.6)	55 (13.1)	126 (13.4)
Casual	27 (5.2)	13 (3.1)	40 (4.2)
Not in workforce	137 (26.2)	81 (19.3)	218 (23.1)
**Occupation**			p = 0.002
Clerical, retail, customer service	89 (17.0)	56 (13.4)	145 (15.4)
Manager	80 (15.3)	71 (16.9)	151 (16.0)
Professional	196 (37.5)	201 (48.0)	397 (42.1)
Other (Tradesmen, labourer, retail)	55 (10.5)	41 (9.8)	96 (10.2)
Not in workforce	103 (19.7)	50 (11.9)	153 (16.2)
**Highest level of education**			0.006
Less than university-educated	166 (31.7)	99 (23.6)	265 (28.1)
University educated	357 (68.3)	320 (76.4)	677 (71.9)
**HIV Status**			0.372
HIV positive	59 (11.3)	39 (9.3)	98 (10.4)
HIV negative	449 (85.9)	372 (88.8)	821 (87.2)
Unknown/untested	15 (2.9)	8 (1.9)	23 (2.4)
**Sexual identity**			0.336
Gay	464 (88.7)	380 (90.7)	84 (89.6)
Bisexual	28 (5.4)	23 (5.5)	51 (5.4)
Other	31 (5.9)	16 (3.8)	47 (5.0)

### Trends in COVID-19 Testing

Almost half of participants (44.5%; N = 419) reported ever testing for COVID-19 with a total of 826 tests among these participants during the study period (mean: 1.034; CI = 1.032–1.036). Most of those who had been tested (98.6%) reported being tested for COVID-19 with a nasal swab. Among those, 53.0% (N = 222) reported 1 test, 20.5% reported 2 tests (N = 90), and 25.5% (N = 107) reported 3 or more tests (range: 3–11). Most (98.6%) reported being tested for COVID-19 through a nose and/or throat swab. Over the study period, reported COVID-19 testing rates in the previous 7 days fluctuated between 0.7% during the week of December 6th 2020 and 12.9% during the week of the 16th May 2021. On average, about 3.3% of the sample reported testing for COVID-19 each week, with increases in testing coinciding with increases of new cases in participants’ state of residence.

**Fig. 1:**
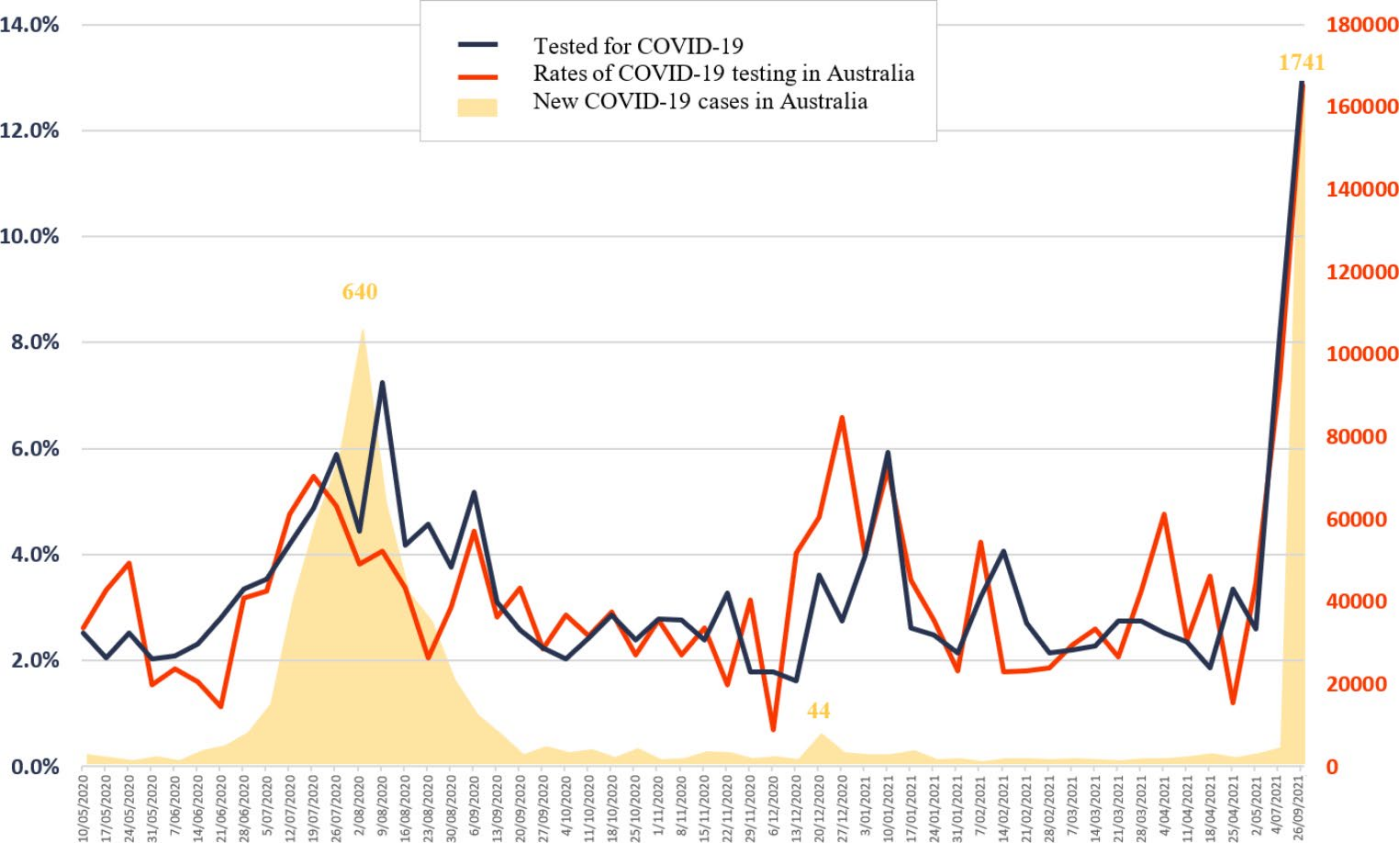
Trends in COVID-19 testing in Australia and among men in this sample, and trends in new COVID-19 notifications in Australia during study period – May 2020 – September 2021 (N = 942)

### Associations with COVID-19 Testing

In bivariable analysis, COVID-19 testing was higher among GBM who were younger (Table 2). For every 1-year increase in age, the rate of testing decreased (HR = 0.990; CI = 0.984–0.996; p = 0.002). In separate analysis, no associations were found between age and avoiding physical contact outside the home, such as avoiding doctors’ offices (p = 0.717) or physical encounters with other gay men (p = 0.274). Men who completed tertiary education were more likely to report testing for COVID-19 compared to men who did not complete any tertiary education (HR = 1.435; CI = 1.137–1.811; p = 0.002). GBM not in the workforce were less likely to report testing for COVID-19 (HR = 0.0.574; CI = 0.407–0.808; p = 0.001).

**Table 2:** Factors associated with testing for COVID-19 (N = 942)

	Hazard Rate Ratio (HR)	95) Confidence interval		p-value	Adjusted hazard Rate Ratio (aHR)	95) Confidence interval		p-value
		**Lower**	**Upper**			**Lower**	**Upper**	
Number of COVID-19 cases reported								
0–10 cases	1				1			
11–100 cases	1.162	0.924	1.462	0.200				
100–1000 cases	1.100	0.762	1.587	0.612				
> 1000	1.553	1.165	2.070	0.003	1.334	1.010	1.761	0.042
Age	0.990	0.984	0.996	0.002	0.988	0.982	0.994	< 0.001
Country of birth								
Australia	1							
New Zealand, North America, and Europe	0.851	0.650	1.112	0.237				
Elsewhere	1.018	0.727	1.427	0.917				
Education								
Less that university educated	1				1			
University educated	1.435	1.137	1.811	0.002	1.314	1.052	1.643	0.016
Employment								
Full time	1							
Part time	0.830	0.618	1.116	0.217				
Casual	0.603	0.140	2.598	0.497				
Not in workforce	0.684	0.533	0.877	0.003				
Occupation								
Clerical, retail, customer service	1							
Manager	0.963	0.686	1.351	0.826				
Professional	1.247	0.956	1.626	0.104				
Other (Tradesmen, labourer, retail)	0.827	0.450	1.521	0.541				
Not in workforce	0.574	0.407	0.808	0.001				
Sexual identity								
Gay/homosexual	1							
Bisexual	0.942	0.616	1.438	0.781				
Other	1.345	0.613	2.953	0.459				
HIV status								
Positive	1							
Negative	1.245	0.915	1.694	0.164				
Unknown/untested	0.644	0.348	1.191	0.161				
Social engagement with other gay men	1.113	1.007	1.231	0.036	2.947	1.562	5.560	0.001
Proportion of gay friends avoiding social events								
A few or some	1							
Most or all	1.465	1.209	1.775	< 0.001				
Proportion of gay friends intending to get vaccinated								
A few or some	1				1			
Most or all	2.211	1.660	2.943	< 0.001	2.028	1.515	2.714	< 0.001
Coping throughout the COVID-19 pandemic								
Coping well	1				1			
Coping poorly	1.420	1.159	1.740	0.001	1.418	1.168	1.721	< 0.001
Mean number of recent sex partners	0.997	0.962	1.033	0.869				
Contact with someone who has COVID-19								
No	1				1			
Yes	2.101	1.367	3.228	0.001	2.281	1.578	3.297	< 0.001
Willingness to contact a non-relationship sexual partner if close contact to COVID-19								
Not at all/somewhat willing	1				1			
Very willing	2.544	1.548	4.180	< 0.001	2.320	1.422	3.785	0.001
Number of people (other than people you live with or your regular partner) in close physical contact.	0.994	0.981	1.007	0.392				

Compared to men who resided in jurisdictions reporting less than 10 new COVID-19 cases in the study week, GBM who resided in jurisdictions reporting more than 1000 new COVID-19 cases in the study week were more likely to report testing for COVID-19 (HR = 1.553; CI = 1.165–2.070; p = 0.002). Compared to men who reported they were coping well in the week in the previous week, men reporting coping poorly were more likely to report testing for COVID-19 (HR = 1.420; CI = 1.159–1.740; p = 0.001).

Having more gay friends was associated with greater likelihood to test (HR = 1.113; CI = 1.007–1.231; p = 0.036). Compared to men whose gay friends did not avoid social events during COVID-19 restrictions, men who reported most or all their gay friends were avoiding social events were more likely to report testing for COVID-19 (HR = 1.465; CI = 1.209–1.775; p < 0.001). Similarly, men who reported most or all their gay friends were intending to be vaccinated were more likely to report testing for COVID-19 than those who reported fewer of their gay friends intending to vaccinate (HR = 2.221; CI = 1.660–2.943; p < 0.001). Most men indicated being “somewhat” (7.4%) or “very” (92.6%) willing to contact their sex partners for contact tracing purposes if they were informed they had been in contact with someone with either COVID-19 or a sexually transmissible infection. Compared to men who were “not at all” or just “somewhat” willing to contact sexual partners, those who were “very” willing were more likely to test for COVID-19 (HR = 2.544; CI = 1.548–4.180; p < 0.001).

In multivariable analysis, compared to living in a state where fewer than 10 new COVID-19 cases within the study week were reported and with all other variables held constant, GBM who resided in a state reporting more than 1000 new cases during the study week were more likely to report testing for COVID-19 (aHR = 1.334; CI = 1.010–1.761; p = 0.042) (Table 2). Younger men were more likely to report COVID-19 testing (aHR = 0.988; CI = 0.982–0.994; p < 0001). Compared to men who reported no tertiary education, GBM who reported tertiary education were more likely to report testing for COVID-19 (aHR = 1.300; CI = 1.096–1.544; p = 0.003). Coping poorly was associated with testing for COVID-19 (aHR = 1.418; CI = 1.168–1.721; p < 0.001), and testing for COVID-19 was associated with greater social engagement with other gay men (aHR = 2.947; CI = 1.562–5.560; p = 0.001) and with reporting that most or all their gay friends intended to vaccinate against COVID-19 (aHR = 2.028; CI = 1.515–2.714; p < 0.001). Compared to men who were not at all or just somewhat willing to contact their sexual partners, those who were very willing to do so were more likely to test for COVID-19 (aHR = 2.320; CI = 1.422–3.785; p < 0.001).

## Discussion

Between May 2020 and September 2021, almost half of GBM in our sample had tested for COVID-19. High rates of testing for COVID-19 in this sample largely reflect high rates of testing in the general population [6–8] (Fig. 1). GBM who reported close contact with someone with COVID-19 were more likely to get tested. GBM residing in the two jurisdictions reporting over 1000 new COVID-19 infections in the study week were more likely to test for COVID-19. These patterns may reflect public health requests for community-wide testing in response to significant outbreaks. In Sydney, for example, city-wide testing vigilance was emphasized, despite the geographic specificity of the outbreak [28]. Of course, there were undoubtedly structural limitations that also applied, thereby effectively capping the maximum number of possible tests that could feasibly be conducted each day.

Factors associated with COVID-19 testing were similar to factors previously found to be associated with HIV testing among GBM [17, 18]. Social contact with others increases the risk and likelihood of potential exposure to COVID-19. Despite this, we found no association between COVID-19 testing practices and the number of persons (outside the participants’ homes) with whom they had close physical contact. Social connectedness generally plays a positive role in fostering health-related help-seeking behaviors, and this has also been the case during the COVID-19 pandemic [14, 15]. Norms within gay community networks played a central role in the response to the HIV epidemic in Australia and in normalizing HIV testing [17, 27, 29]. This also appears to apply to testing for COVID-19 among GBM in our sample, where testing was associated with greater social engagement with gay men, and with having a greater proportion of gay friends who engaged in practices intended to minimize COVID-19 transmission. Just as peer networks have played an important role in disseminating information about, and normalizing, new HIV prevention technologies throughout the HIV epidemic [18, 23, 30], so too might community networks provide a key locus for the promotion of COVID-19 health-related help-seeking behaviors.

Despite the greater impact of COVID-19 on older cohorts, older GBM in our analysis reported lower testing rates for COVID-19. Given older age has been associated with greater engagement with health care and with being more socially connected to gay communities [29], this was surprising. Lower testing rates among older GBM could suggest they were avoiding social contact throughout the pandemic and thereby negating the need for COVID-19 testing. However, we found no association between age and physical distancing practices. Alternatively, younger men tend to have more mobile occupations with greater exposure to the public, such as hospitality, customer service, and delivery, possibly necessitating more testing.

Higher reported cases of COVID-19 reflected an increased prevalence of participants self-reporting coping poorly. This peaked during periods coinciding with highest numbers of COVID-19 tests performed, and the substantial Australian outbreaks (August through September 2020, and January and September 2021). Following the suppression of these outbreaks and while new COVID-19 cases remained low, there was a downward trend in participants reporting that they were coping poorly. Increases in COVID-19 testing among GBM who were coping poorly were likely attributable to elevated fears of COVID-19 infection and the anxieties that accompany physical distancing requirements.

Most men in our sample indicated a willingness to cooperate with contact tracing if required for COVID-19 control, as was also demonstrated in a significant outbreak in the United States within tight social networks of GBM in Provincetown, Massachusetts during 2021 [31]. On that occasion, it was acknowledged by staff from the United States Centers for Disease Control that the supportive role of gay community networks played a key role in understanding and addressing that particular outbreak. So, it would appear that peer networks among GBM in our sample also play an important role in disseminating health information and perhaps even normalizing COVID-19 testing in similar ways [15, 16].

### Limitations

Our study used an online convenience sample and compared with other samples of Australian GBM, ours was older, more educated, and included relatively few bisexual and overseas-born men [22]. Participants may have also been more inclined to participate due to disproportionate concern over COVID-19. Given this, findings may not be representative of all GBM. Due to the rapid onset of COVID-19 and the need to implement rapid monitoring, some of the measures used had not been previously tested. Although long-term characteristics could have influenced the likelihood of COVID-19 testing, this study was not set up to investigate the long-term influences on COVID-19-related behaviors. In terms of social networks, participants were only asked about connections to other gay men, so the role of other peers and social contacts in supporting testing was not assessed. There may be features of gay social networks which are unique in encouraging testing and engagement with health services, but our measure of social engagement with gay men may simply be a proxy for participants who had larger, more supportive social networks.

## Conclusion

Social connection with other gay men was associated with COVID-19 testing, similar to what has been observed during the HIV epidemic, making community networks a potential focus for the promotion of COVID-19 safe practices. Ongoing investigations will determine the short-term impacts of the COVID-19 pandemic on vaccinations and other health-related help-seeking behaviors, as well as any sustained long-term effects.
